# Nonlinear Absorption and Refraction of Picosecond and Femtosecond Pulses in HgTe Quantum Dot Films

**DOI:** 10.3390/nano11123351

**Published:** 2021-12-10

**Authors:** Arturs Bundulis, Ivan A. Shuklov, Vyacheslav V. Kim, Alaa A. Mardini, Jurgis Grube, Janis Alnis, Anna A. Lizunova, Vladimir F. Razumov, Rashid A. Ganeev

**Affiliations:** 1 Institute of Solid State Physics, University of Latvia, Kengaraga 8, LV-1063 Riga, Latvia; arturs.bundulis@cfi.lu.lv (A.B.); jurgis.grube@cfi.lu.lv (J.G.); 2 Moscow Institute of Physics and Technology, National Research University, Dolgoprudny 141701, Russia; Aladdin.mardini@phystech.edu (A.A.M.); lizunova.aa@mipt.ru (A.A.L.); razumovvf@list.ru (V.F.R.); 3 Laboratory of Nonlinear Optics, University of Latvia, Jelgavas 3, LV-1004 Riga, Latvia; vyacheslav.kim@lu.lv; 4 Department of Chemistry, Faculty of Science, Damascus University, Damascus P.O. Box 30621, Syria; 5 Quantum Optics Laboratory, Institute of Atomic Physics and Spectroscopy, University of Latvia, Jelgavas 3, LV-1004 Riga, Latvia; janis.alnis@lu.lv; 6 Institute of Problems of Chemical Physics, Russian Academy of Sciences, Chernogolovka 142432, Russia; 7 Department of Physics, Voronezh State University, Voronezh 394006, Russia

**Keywords:** quantum dots, mercury telluride, thin films, saturable absorption, nonlinear refraction

## Abstract

We report measurements of the saturated intensities, saturable absorption, and nonlinear refraction in 70-nm thick films containing 4 nm HgTe quantum dots. We demonstrate strong nonlinear refraction and saturable absorption in the thin films using tunable picosecond and femtosecond pulses. Studies were carried out using tunable laser pulses in the range of 400–1100 nm. A significant variation of the nonlinear refraction along this spectral range was demonstrated. The maximal values of the nonlinear absorption coefficients and nonlinear refractive indices determined within the studied wavelength range were −2.4 × 10^−5^ cm^2^ W^−1^ (in the case of 28 ps, 700 nm probe pulses) and −3 × 10^−9^ cm^2^ W^−1^ (in the case of 28 ps, 400 nm probe pulses), respectively. Our studies show that HgTe quantum dots can be used in different fields e.g., as efficient emitters of high-order harmonics of ultrashort laser pulses or as laser mode-lockers.

## 1. Introduction

Small-sized agglomerates of mercury telluride (HgTe) have attracted attention as efficient materials for mid-IR applications [1,2,3,4,5,6,7,8]. The low-order optical nonlinearities of mercury telluride quantum dots (MTQDs) may also attract interest in future due to the connection between them and higher-order nonlinear optical response. In particular, one would expect to observe a direct relation with the generation efficiency of high-order harmonics (HHG) in the medium containing the quantum dots (QDs) possessing strong nonlinear refraction and absorption.

One can assume that if the low-order, nonlinear optical properties of MTQDs, e.g., nonlinear refraction (NR) and nonlinear absorption (NA), become close to those of Ag_2_S, ZnS, Cd_0.5_Zn_0.5_S QDs, demonstrating a more efficient generation of harmonics compared to species like the InP@ZnS core-shell QDs and HgSe QDs (which show low conversion efficiencies during HHG), then a large harmonic yield will occur from mercury telluride QDs. It is worth noting that previous studies of the former group of QDs have demonstrated notable enhancements of harmonic yield compared with when HHG is carried out in plasma produced on the surfaces of bulk silver sulfide, zinc sulfide, or alloyed cadmium-zinc sulfide [9,10]. Similarly, the latter group of QDs (InP@ZnS and HgSe) demonstrating relatively weak NR and/or NA gives rise to high conversion efficiencies of the harmonics of ultrashort pulses.

Previously, MTQDs were not used as the medium for HHG. Rather, these QDs were typically used for the detection of low power IR emissions. Confirmation of above assumption will support the conclusion about the connections among different types of optical nonlinearities. It is worth noting that even as a medium which demonstrates a moderate level of HHG efficiency, these MTQDs will be more useful compared with molecular/ionic HgTe species or large HgTe nanoparticles. This statement is not an assumption, but rather, a rule confirmed during numerous plasma and gas HHG experiments when small-sized agglomerates demonstrated a larger harmonic yield compared with the molecular species of the same elemental consistency.

The usefulness of these species, apart from IR applications [1,2,3,4,5,6,7,8], is not limited to potential use in HHG. Other applications of the colloidal suspensions of MTQDs may include mode-lockers or Q-switchers in lasers, due to their strong saturable absorption (SA). Another possibility is the application of their strong reverse saturable absorption (RSA) at relatively high laser intensities in optical limiting devices for the protection of the sensitive registrars and eyes.

Altering the nonlinear refractive index (*γ*) and nonlinear absorption coefficient (*β*) of these QDs makes it possible to measure them using nanosecond, picosecond and femtosecond probe pulses (PP). The analysis of NR, SA, and RSA using pulses of different durations could reveal differences among those processes in MTQDs.

To clarify the potential advantages of these QDs for the generation of coherent extreme ultraviolet emission during propagation of femtosecond pulses through the plasmas produced on surfaces containing MTQDs, one has to analyze the low-order nonlinearities of those species using short (i.e., picosecond and/or femtosecond) laser pulses. To the best of our knowledge, there are no published results on the nonlinear optical properties of MTQD films using short laser pulses. In this paper, we report on the synthesis and determination of the saturated intensity, saturable absorption, and nonlinear refraction during propagation of 28 ps and 150 fs pulses through 70-nm-thick films containing 4-nm mercury telluride quantum dots. We analyze the wavelength-dependent variations of the above processes using tunable laser sources in the range of 400–1100 nm.

## 2. Characterization of MTQDs

Synthesis of mercury telluride with narrow size-distribution was performed by the hot-injection organometallic method. In this procedure, we used a tellurium precursor prepared from *n*-trioctylphosphine and tellurium [11,12]. Reaction of this precursor with mercury halides requires significantly lower temperatures (60–80 °C) than with lead or cadmium for the preparation of the corresponding tellurides [13]. Small HgTe CQDs capped with 1-dodecanethiol (DDT) were obtained with short reaction times and temperatures as low as 60 °C. The obtained colloidal solutions demonstrated good colloidal stability.

In the typical synthesis of MTQDs, two precursor solutions were prepared:

Solution A: 6 mL of dry oleylamine was added to 60 mg of HgCl_2_ in two round bottom flasks equipped with septa in argon at room temperature. The resulting solution was gradually heated to 100 °C for 1 h under Ar.

Solution B: 0.4 mL of 1M tellurium in tri-n-octylphosphine (TOP) was added to 2 mL dry oleylamine under argon and gradually heated to 100 °C for 1 h.

Then, the solutions were cooled to 60 °C and solution B was rapidly injected by syringe into solution A under continuous argon flow. The mixture was stirred for 90 min at 60 °C. The reaction was quenched by adding a mixture of 16 mL tetrachloroethylene (TCE), 3.1 mL (0.8 mmol) DDT and 1.5 mL TOP, followed with fast cooling using an ice bath.

HgTe nanocrystals were isolated by adding 5 mL of methanol per 7.5 mL of the resulting solution, followed by centrifugation. The resulting precipitate was redispersed in 2.5 mL of TCE, and 2.5 mL acetonitrile was added to the resulting solution, which was centrifuged once again. The precipitate was dried under Ar and redispersed in 0.5 mL of TCE. Finally, the resulting solution was filtered using a 0.22 µm hydrophobic polytetrafluoroethylene syringe filter.

**Figure 1:**
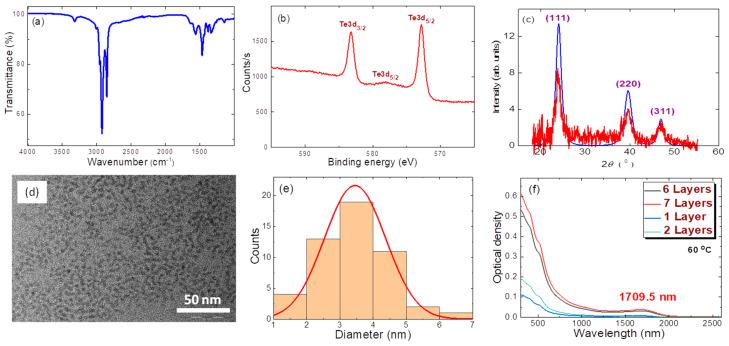
(**a**) FTIR spectra in the range of 5000–800 cm^−1^. (**b**) High-resolution XPS spectrum. (**c**) X-ray diffraction image. Blue curve: calculation. Red curve: experiment. (**d**) TEM image of MTQDs. (**e**) Histogram of particles distribution. (**f**) Vis-IR absorption spectra of thin films.

The mean size of the studied QDs was 4 nm (a morphological characterization is given below). The optical absorption of the prepared QD suspension showed stronger absorbance at the 500 nm compared with the 1000 nm region. The details of the optical absorbance of films will be discussed below. The ligand shell was analyzed in the infrared range (5000–800 cm^−1^) using Fourier-transform infrared spectroscopy (FTIR, Spectrum 100, Perkin Elmer, Waltham, MA, USA), and the presence of 1-dodecanethiol was confirmed by FTIR. C–H stretching of methyl and methylene groups could be observed at 2924 cm^−1^ and 2850 cm^−1^, and bending of methylene groups was seen at 1465 cm^−1^. Free thiol was not observed in FTIR spectrum (Figure 1a).

The composition of the studied MTQDs was carefully analyzed, since they could potentially undergo oxidation in air. Oxidized QDs should show a deviation from the reported X-ray photoelectron spectroscopy (XPS), X-ray diffraction (XRD), FTIR and other spectra of pure MTQDs. Below, we describe the procedure to confirm the presence of the latter species during the nonlinear optical experiments.

The chemical composition of the nanocrystals was explored by XPS. XPS measurements (PHOIBOS 150 MCD, SPECS Surface Nano Analysis GmbH, Berlin, Germany) were performed using Mg Ka radiation (1253.6 eV). High-resolution XPS spectrum (Figure 1b) confirmed mercury telluride as the main constituent of the monocrystalline core [14]. The obtained XPS spectrum with characteristic binding energy values of 583.2 eV (Te 3d_3/2_) and 572.8 eV (Te 3d_5/2_) was consistent with the known Te^2−^ spectra. The crystal structure was identified by XRD (ARL X’TRA X-ray powder diffractometer, Thermo Fisher Scientific Inc., Berlin, Germany) using CuKa radiation. XRD (Figure 1c) confirmed the zinc blende structure of HgTe colloidal QDs. The XRD spectra of MTQDs demonstrated a single crystalline phase. Previous studies have used XRD to examine the oxidation of HgTe [15]. Such samples are characterized by tellurium oxidation products. Mercury tellurite (HgTeO_3_) and paratellurite (TeO_2_), which are typical for heavily oxidized HgTe samples, were absent in our XRD spectra. Our investigation did not include study of the luminescence properties of the HgTe samples. Nevertheless, based on XPS and XRD data, as well as FTIR measurements, we can exclude heavy or significant oxidation of our samples.

Transmission electron microscopy (TEM JEM-2100, JEOL GmbH, Freising, Germany) revealed a mean diameter of 4 nm (Figure 1d,e) that fit well with the observed first absorption peak in the range of ~1570 nm in the TCE solution, according to previously reported data on spherical HgTe nanoparticles. The preparation of thin films was carried out by layer-by-layer deposition using dip-coating on a glass substrate. Exchange of ligand shell by 1,2-ethanedithiol (EDT) was performed using 1:1:100 EDT/HCl/2-propanol solution. The glass was withdrawn at a speed of 30 mm/min. Excess exchanging reagent was removed by washing the thin films in 2-propanol. The process was repeated 5 to 20 times in order to obtain films with thicknesses of 50 nm or more. The spectrum absorption band was shifted toward the longer wavelength range. Figure 1f shows the absorption spectra of a few thin films and the appearance of a peak at around 1710 nm after ligand exchange.

Details of the preparation of the MTQD colloidal suspensions and multilayer films are presented in Table 1 and Table 2.

## 3. Experimental Arrangements and Fitting Procedure for Nonlinear Optical Studies

**Figure 2:**
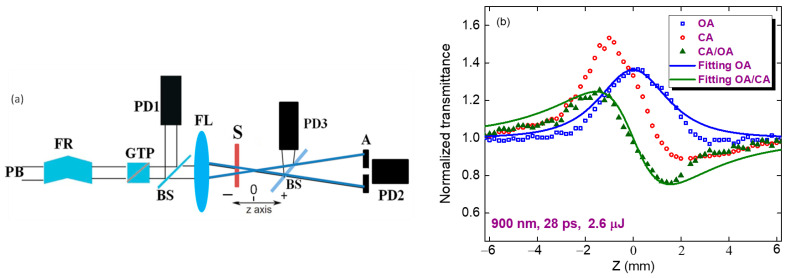
(**a**) Experimental setup for the nonlinear optical studies using the Z-scan scheme. PB: probing beam; FR: Fresnel rhomb; GTP: Glan-Taylor prism; BS: beamsplitter; FL: focusing lens; S: sample; A: aperture; PD1-PD3: photodiodes for determination of normalized transmittance, nonlinear refraction, and nonlinear absorption. (**b**) Z-scans of MTQD film using 900 nm, 28 ps pulses: closed-aperture scan (CA, red empty circles), open-aperture scan (OA, blue empty squares), and CA/OA division (green filled triangles). Solid curves represent the fittings using Equations (1) (green curve) and (2) (blue curve).

The standard Z-scan technique was used to study the nonlinear optical properties of the MTQD films (inset in Figure 2). Laser radiation was focused with a 110 mm focal length spherical lens. The sample (70-nm film of MTQD deposited on a 1-mm thick silica glass plate) was moved along the z axis through the focal plane of the spherical lens. The propagated radiation was measured by PD3 (open-aperture (OA) scheme) and PD1 (closed-aperture (CA) scheme) photodiodes. PD1 was used to determine the normalized transmittances in the case of OA and CA Z-scan schemes. Two tunable laser sources (EKSPLA PG400 + PL2210 picosecond laser and ORPHEUS-HP + PHAROS PH2 femtosecond laser) were used in this process. The use of 28 ps pulses with a 1 kHz repetition rate and 150 fs pulses with a 500 kHz repetition rate allowed us to tune along the 400–1200 nm spectral region.

The most manifested processes were the SA and NR in the case of picosecond pulses, and SA, RSA, and NR in the case of femtosecond pulses. The fitting of the experimental CA data was carried out using the following equation [16]:

(1) $$T=1+\frac{2(-\rho x^{2}+2x-3\rho )}{\left(x^{2}+9)(x^{2}+1\right)}\Delta \Phi_{o}$$

Here, *x = z*/*z*_0_, *z*_0_ is the Rayleigh length of the focused radiation, *z*_0_ = π(*w*_0_)^2^/*λ*, *w*_0_ is the beam waist radius, *λ* is the wavelength of the probe radiation, *ρ = β*/2*kγ,* *k =* 2π/*λ,* ΔΦ_o_ = *k*γ*I*_0_*L*_eff_, *I*_0_ *is* the laser radiation intensity in the focal plane, *L*_eff_ = [1 − exp *(*−*α*_0_*L*)]/*α*_0_ is the effective length of the sample, *α*_0_ is the linear absorption coefficient, and *L* is the thickness of the studied film.

To fit the OA data and determine the nonlinear absorption coefficient responsible for SA (*β*_SA_), we used the following equation [17]:

*T* ≈ 1 − *p*(*x*)/2(2)^1^^/2^, (2)

where *p*(*x*) = *β*_SA_*I*_0_*L*_eff_/[1 *+ x*^2^].

We also determined the saturation intensity (*I*_sat_), which was defined as the optical intensity required for a two-fold increase in normalized transmittance in the case of SA.

## 4. Nonlinear Optical Characterization of MTQD Films Using Tunable Picosecond Pulses

The 70-nm thick films produced with a slow dip-coating speed were used in these studies. The NA around the whole spectral range was entirely determined by SA (see, for example, Figure 2, blue squares in the case of 900 nm PP). The *γ* was determined from the fitting of the curve obtained after division of the CA data by the OA data. The results are shown as green triangles. The peak-valley configuration of this curve revealed that the nonlinear refractive index was negative, which showed that self-defocusing occurred in the studied films when they were irradiated by moderately intense (3 × 10^9^ W cm^−2^), 900 nm laser pulses.

The negative NR of the films in the case of 900 nm picosecond PP indicates the prevailing influence of Kerr-related self-defocusing (green filled triangles showing CA/OA scan). The solid curves in Figure 2a show the CA and OA fittings using Equations (1) (green curve) and (2) (blue curve). The *γ* of the studied film was calculated to be −8 × 10^−10^ cm^2^ W^−1^ in the case of 900 nm, 28 ps PP.

In accordance with the model of saturated absorption, the relaxation rate of excitations does not depend on the intensity [18]. The absorption rate A was determined by A = *α*_0_ /(1 + *I*/*I*_sat_). Here, *I* is the variable intensity along the Z-axis during focusing of the laser radiation. *α*_0_ and *I*_sat_ are related to the concentration N of the active centers in the medium, the effective cross-sections σ and the lifetime τ of the excitations. An OA Z-scan of 70 nm thick films (Figure 2, blue empty squares) showed bleaching at *λ* = 900 nm and *I*_0_ = 3 × 10^9^ W cm^−2^ with *β*_SA_ = −1.4 × 10^−5^ cm W^−1^. The corresponding saturated intensity was determined to be *I*_sat_ = 7 × 10^9^ W cm^−2^. No positive NA was observed in the CA/OA curve, which was almost symmetric with regard to the *T* = 1 line. The fitting parameter *ρ* was less than 10^−3^, which corresponded to *β* < 10^−7^ cm W^−1^, i.e., three orders of magnitude less than the SA-related absorption.

**Figure 3:**
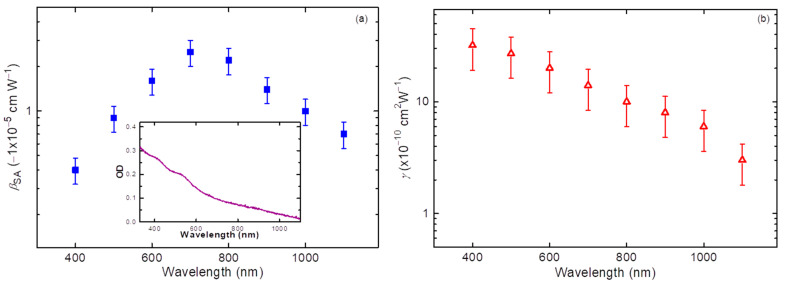
Spectral dependences of the negative nonlinear absorption attributed to SA (**a**) and the negative nonlinear refraction (**b**) of the MTQD film measured by 28 ps probe pulses. Inset in (**a**): optical density (OD) of the film in the studied spectral region. Our film was characterized by steady growth of absorbance toward shorter wavelengths.

Similar measurements were carried out in the broad spectral range of PP (Figure 3) with 100-nm steps of increasing wavelength. The optical density of the film in the 330–1100 nm range is shown in the inset to Figure 3a.

SA was the dominant nonlinear optical effect in this wavelength range. The maximal value of *β*_SA_ was determined to be −2.5 × 10^−5^ cm W^−1^ at *λ* = 700 nm. The spectral dependence of this parameter is shown in Figure 3a. In a shorter wavelength region (*λ* < 700 nm), a steady decrease of the nonlinear absorption coefficient attributed to SA was observed. SA does not directly follow the spectral absorption dependence, since the former process depends on different factors related to the structure of the energy levels of the studied material. Such saturable absorbers can be useful in laser cavities for passive Q-switching and mode-locking. Thus, the studied thin QD films could be applied as Q-switchers for semiconductor, solid-state, and dye lasers irradiated in the 700–800 nm range. In particular, SA is only one of several mechanisms that produce self-pulsation in lasers, especially in semiconductor lasers.

NR was observed across the whole region of the used wavelengths. Figure 3b shows the spectral dependence of *γ* in the 400–1100 nm region. NR steadily increased with the decrease of the PP wavelength.

## 5. Nonlinear Optical Characterization of MTQD Films Using Tunable Femtosecond Pulses

**Figure 4:**
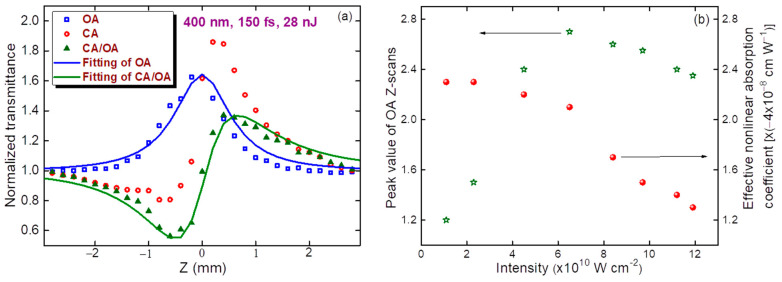
(**a**) Z-scans of MTQD film using 400 nm, 150 fs pulses: closed-aperture scan (CA, red empty circles), open-aperture scan (OA, blue empty squares), and CA/OA division (green filled triangles). Solid curves represent the fittings using Equations (1) (green curve) and (2) (blue curve). (**b**) Dependences of the peak value of the OA Z-scan (green stars) and effective nonlinear absorption coefficient (red diamonds) on the intensity of 400 nm, 150 fs pulses.

CA and OA scans using femtosecond pulses are shown in Figure 4a for the following PP conditions: *λ* = 400 nm, *t* = 150 fs, *E* = 28 nJ, and *I*_0_ = 6 × 10^10^ W cm^−2^. As in the case of picosecond PP, the dominant mechanism here was SA, although self-defocusing was replaced by self-focusing. The difference in the focal spot sizes of the picosecond and femtosecond pulses (32 and 20 µm (FW1/e^2^M)) gave rise to differences in the Rayleigh length z_0_ (2 and 0.7 mm, respectively). These parameters approximately corresponded to the relation between the measured valley-to-peak distance in the CA Z-scans and z_0_ (ΔZ ≈ 1.7z_0_) [17]). The corresponding values of ΔZ in the case of picosecond and femtosecond pulses were 3.4 mm (Figure 2) and 1.2 mm (Figure 4a), respectively. Thin sample approximation was maintained in these experiments, because the width of the studied films (70 nm) was less than the Rayleigh lengths of the applied beams.

The *γ* of the studied film at this wavelength with femtosecond pulses was shown, from the fitting of the CA/OA Z-scan (Figure 4a, solid green curve), to be 3 × 10^−11^ cm^2^ W^−1^. This parameter significantly dropped with increasing PP wavelength. At 600 and 650 nm, the CA Z-scans did not show nonlinear refraction, but rather, revealed mostly SA. Under these conditions, growth of the pulse energy above 100 nJ led to some irregularities, which were attributed to the scattering induced by the modification of the film. We did not see the appearance of holes. However, the nonlinear scattering at high intensities can modify the nonlinear optical response of the film.

*β*_SA_ and *I*_sat_ were calculated using the fitting shown by the blue solid curve in Figure 4a (−1 × 10^−7^ cm W^−1^ and 9 × 10^10^ W cm^−2^, respectively). In this case, we also observed a positive NA (see the asymmetric CA/OA scan), which was calculated by determining parameter *ρ* = *β*/2*kγ* (*β* = 2.3 × 10^−8^ cm W^−1^). The intensity dependence of the peak variations of the SA allowed us to define the energy and intensity range at which the steady growth of the former parameter became suppressed by the involvement of the opposite process of the positive nonlinear absorption. Figure 4b shows this dependence up to the *I*_0_ = 1.2 × 10^11^ W cm^−2^ in the case of 400 nm PP. A similar dependence of the nonlinear absorbance on the intensity of the PP was observed throughout the entire tunable femtosecond radiation spectrum (400–1100 nm). With high intensity pulses (>1.5 × 10^11^ W cm^−2^), the nonlinear scattering could decrease the propagation of the on-axis beam.

**Figure 5:**
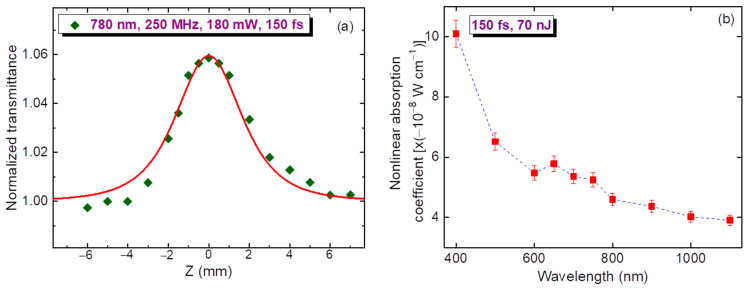
(**a**) OA Z-scan of MTQD film using a high-pulse rate laser (780 nm, 250 MHz, 180 mW, 150 fs). Green rhombs: experiment, solid red curve: fitting using Equation (2). (**b**) Spectral dependence of the nonlinear absorption coefficient *β*_SA_ of thin film. The measurements were carried out using 150 fs, 50 nJ pulses.

We also used a high-pulse-repletion IR laser (250 MHz, 150 fs, 780 nm, 180 mW) for the Z-scans to analyze the heat-related effects. Small energy of pulses (0.7 nJ) did not allow us to observe the Kerr-related NR in this spectral region. We also did not see thermal lens induced self-defocusing in this film. Meanwhile, the SA was still observable under these conditions (Figure 5a).

The spectral dependence of the *β*_SA_ of thin film (under the condition of *I*_0_ = 4.5 × 10^10^ W cm^−2^) on the absence of additional effects like positive NA and nonlinear scattering is shown in Figure 5b. We observed the steady growth of this parameter with a decrease of PP wavelength. At larger PP intensities, this dependence was less pronounced, with smaller relative growth of *β*_SA_ at shorter wavelengths compared with the ~1000 nm region. No RSA was observed under these conditions.

## 6. Discussion

The *γ* and *β*_SA_ of the studied MTQD film are summarized in Table 3. Thin films containing HgTe QDs showed relatively low values of saturated intensities (7 × 10^9^ and 4 × 10^10^ W cm^−2^ in the case of 28 ps and 150 fs pulses, respectively). Low-*I*_sat_ saturable absorbers may allow the formation of low-power femtosecond laser sources. The saturation intensity of light at which the extinction coefficient is reduced by a factor of 2 is an important parameter for such lasers. Among the quantum dots used for modern, passively mode-locked and Q-switched lasers [19,20,21], the studied MTQDs possessed smaller *I*_sat_.

Below we address the differences in the nonlinear refractive indexes of the studied film while using 28 ps and 150 fs PP. The application of the latter, even at a high repetition rate (500 kHz), did not reveal the formation of thermal lenses, resulting in the observation of self-defocusing in the shorter-wavelength region (400–500 nm). It is worth noting that in this region, the linear absorption was relatively large (OD = 0.45; see Figure 1f). Nevertheless, the thin film did not form an accumulative thermal lens. In the case of picosecond pulses, self-defocusing was observed throughout the studied spectral range (400–1100 nm, Figure 3b). In the case of picosecond pulses, the molecular Ker-related nonlinearities of chalcogenides can prevail over electronic Kerr-related processes. Commonly, the former refractive nonlinearities demonstrate self-defocusing properties. Probably, the prevalence of molecular nonlinearities related with reorientational mechanisms, which play an important role in the picosecond timescale over the Kerr-related electronic nonlinearities, gave rise to the observation of the negative sign of the effective nonlinear refractive index in our experiments with 28 ps PP.

One important feature of small-sized species is their variable response related to the size effect; this means that quantum confinement starts playing a decisive role when local-field-related effects significantly enhance the nonlinear optical response of the particles with radii below the Bohr radius of the studied material. This is an interesting phenomenon; however, we did not analyze the nonlinear optical parameters of large particles, since the goal of this work was to synthesize the smallest possible (~3–4 nm) species and measure their nonlinear optical response along a broad spectral range using pulses of different duration.

The choice of MTQDs was justified by their excellent morphological characteristics, e.g., size distribution and small mean size, making it likely that we would observe strong confinement effects on the nonlinear optical properties of the QDs. As Figure 1 depicts, we analyzed the morphological, spectral, stability, etc. features of different synthesized MTQDs, and then analyzed the saturation and refraction nonlinearities of the smallest sample. We would like to underline the fact that the above parameters are among the highest reported so far for small-sized samples.

The relation between the saturated intensity and the electronic structure of HgTe may be as follows. One can attribute the strong saturable absorption observed with this material to its peculiar electronic band structure. We therefore expected a relation between the low threshold for saturable absorption in such a nanoscale material and the exotic electronic structure characterized by a low density of electronic states at the Fermi level. Probably, such a relation could be attributed to MTQDs.

It has been suggested that these nanostructures undergo significant quantization of electron energy levels that can realize some four-level scheme, thereby increasing saturation through the prolonged excited state of electrons [22]. In well-dispersed MTQDs, the biexciton lifetime increases approximately linearly with particle volume, confirming trends observed in other systems. Another study [5] discussed the low threshold gain for spontaneous emission in these species. The authors of that study identified long-lived population inversion that could influence the saturation of absorption in these species, since stimulated emission involves multifold degenerate band-edge states. Correspondingly, the population inversion can be attained only with high pump power, and must compete with an efficient, multiexciton recombination. Probably, the long-lived population inversion plays a role in the strong saturable absorption of MTQDs.

Finally, we briefly address the applicability of the studied species for HHG. Obviously, there are no strict criteria for how to evaluate different QDs as emitters of coherent light in the extreme ultraviolet region. In the present work, we were focused on the study of the nonlinear refractive indices and saturable absorption of MTQDs, rather than on the HHG in this species. However, some relations between those two effects might exist due to the correlation between the lower-order nonlinear optical properties of the studied QDs and their ability to generate strong harmonics, as has been demonstrated with some metal sulfide QDs [9,10]. Therefore, HgTe QDs can be considered as a promising material for the generation of intense harmonics in the extreme ultraviolet range.

## 7. Conclusions

These studies were carried out using picosecond and femtosecond laser pulses which were tunable in the range of 400–1100 nm. The maximal nonlinear absorption coefficients and nonlinear refractive indices determined throughout the studied wavelength range were −2.4 × 10^−5^ cm^2^ W^−1^ (in the case of 28 ps, 700 nm probe pulses and −3 × 10^−9^ cm^2^ W^−1^ (in the case of 28 ps, 400 nm probe pulses), respectively. This study has shown that HgTe quantum dots can be used in different fields, e.g., as efficient emitters of high-order harmonics in ultrashort laser pulses, or as mode-lockers for lasers.

## Figures and Tables

**Table 1:** Parameters of the preparation of MTQD colloidal suspensions. λ_Abs_ is the wavelength of maximal absorbance in the IR range, HgTe QDs (M) is the molar concentration, HgTe QDs (QDs/mL) is the number of QDs in 1 mL.

Reaction Temperature (°C)	Reaction Time (min)	Amount of HgCl_2_ (mmol)	Amount of TOPTe (mmol)	DDT (mmol)	λ_Abs_ (μm)	HgTe QDs (M)	HgTe QDs (QDs/mL)
60	90	0.2	0.2	0.8	1.57	5 × 10^−7^	3 × 10^14^

**Table 2:** Parameters of deposited MTQDs films. λ_Abs_ is the wavelength of maximal absorbance in the IR range, OD is optical density of film, HgTe QDs (M) is the molar concentration, HgTe QDs (QDs/mL) is the number of QDs in 1 mL, Solvent for deposition is TCE.

No. of Layers	λ_Abs_ (nm)	OD	HgTe QDs (M)	[HgTe (QDs)] (QDs/mL)
1	1678.5	0.003	8 × 10^−9^	5 × 10^12^
2	1676.5	0.006	15 × 10^−9^	9 × 10^12^
6	1709	0.018	46 × 10^−9^	28 × 10^12^
7	1709.5	0.021	54 × 10^−9^	32 × 10^12^

**Table 3:** The nonlinear optical parameters of the MTQD film.

	28 ps Pulses		150 fs Pulses	
*λ* (nm)	*γ* [×(−10^−10^ cm^2^ W^−1^)]	*β*_SA_ [×(−10^−5^ cm W^−1^)]	*γ* [×(10^−11^ cm^2^ W^−1^)]	*β*_SA_ [×(−10^−8^ cm W^−1^)]
400	30	0.4	3	10
500	26	0.9	0.7	6.5
600	20	1.7	<0.15	5.2
700	13	2.4		5.1
800	10	2		4.7
900	8	1.4		4.5
1000	6	1		4
1100	3	0.8		3.8

## Data Availability

Data can be available upon request from the authors.
